# Differential attitudes and outcomes of endodontics education between mainland and non-mainland chinese students during COVID-19 pandemic

**DOI:** 10.1186/s12903-023-02901-7

**Published:** 2023-04-06

**Authors:** Ting Zhong, Chufang Liao, Haishan Shi

**Affiliations:** School of Stomatology, Jinan University, Guangzhou, 510632 China

**Keywords:** Dental education, COVID-19, Non-mainland China, Endodontics, Attitudes

## Abstract

**Background:**

The COVID-19 pandemic has changed the learning style and campus life of dental students. This study aimed to evaluate the learning attitudes and outcomes of endodontics among mainland Chinese students and non-mainland Chinese students (students from Hong Kong, Macao, and Taiwan) during the pandemic.

**Methods:**

A cross-sectional survey was conducted in November 2022 at the School of Stomatology, Jinan University, utilizing a self-report online questionnaire, including demographic characteristics and attitudes toward the endodontic course and the COVID-19 pandemic. The endodontics scores were collected from recruited students for further analysis. The collected data were analyzed using SPSS 22.0 software, with independent two-sample t-tests to compare continuous variables and chi-square tests for categorical variables.

**Results:**

A total of 215 dental students completed the survey, with 126 (58.6%) of them being non-mainland Chinese students. Compared to mainland Chinese students, non-mainland Chinese students had lower scores in both theoretical (63.6 ± 13.5 vs. 83.2 ± 8.00) and skill (88.4 ± 5.38 vs. 90.0 ± 4.91) endodontic assessments. Non-mainland Chinese students reported significantly greater impacts of the COVID-19 pandemic on their learning emotions, personal hygiene, and future career choices compared to mainland Chinese students.

**Conclusions:**

Non-mainland Chinese students had poorer academic performance in endodontics and experienced a greater impact from the COVID-19 pandemic in terms of their studies and lives. Dental educators should consider the diversity of students and take necessary measures to support their mental health and enhance learning outcomes in the post-COVID-19 era.

## Background

The coronavirus disease-19 (COVID-19) pandemic, which has now ongoing for over 3 years, remains an acute global emergency. In the week of 2 to 8 January 2023, almost 2.9 million new cases and over 11,000 deaths were reported worldwide, with a total of 659 million confirmed cases and 6.6 million deaths from COVID-19, as reported by World Health Organization [1]. To combat the spread of the virus, maintaining social distance has become a critical rule in all countries, impacting all aspects of human life on a global scale [2]. In response, the Chinese government implemented rapid and comprehensive public health emergency interventions [3], such as closing schools and public places, and encouraging social distancing among students [4]. However, despite easing epidemic prevention and control measures since December of last year, the COVID-19 pandemic continues to unfold [5]. There seems to be no doubt that campus students could not resume normal life as before the pandemic in the short term.

The pandemic has had far-reaching implications for education systems worldwide, with the closure of universities and borders in many countries severely impacting higher education [6, 7]. Among the fields significantly affected by the pandemic is the education of future dental professionals [8]. Imposition of unfamiliar public health measures, including social distancing, social fear, and closed campus, have negative psychological impacts on dental students [9]. Although distance learning become a widely adopted strategy for higher education in various fields, the quality of teaching and learning remains a concern. Besides, dental education faces a unique challenge due to its reliance on clinical experience to achieve minimum competency in performing dental treatments. Since many dental procedures produce considerable amounts of aerosols and droplets, many dental treatments were suspended during the pandemic, thus affecting the intern and training of dental students [10]. Hence, COVID-19 has had a profound impact on the lives and studies of dental students.

Jinan University is the first overseas Chinese school founded by the Chinese government, which has been recruiting students from Hong Kong, Macau, Taiwan, and overseas. Notably, these students account for about 60% of the medical student population. These regions possess distinct advantages in terms of education resources, economic and trade environment, and industrial foundation when compared to mainland China [11]. Furthermore, the primary and secondary school education in these regions is more comparable to that of western developed countries [12]. The dissimilarities in prior educational backgrounds between students from Hong Kong, Macao, Taiwan (abbreviated as non-mainland Chinese students) and mainland Chinese students result in distinct learning abilities in the university setting [13]. A better understanding of the relations between mainland Chinese and non-mainland Chinese students, as well as their adaptation to study in mainland China, is critical to the formulation and delivery of policies and program designed to support their mental health and educational outcomes [14].

Endodontics is the most basic and important course of dentistry that deals with the diagnosis and treatment of oral conditions which arise as a result of pathosis of the dental pulp and peri-radicular tissues [15]. In the endodontic course, it is great important for students to master both the theoretical knowledge and experimental skills [16]. During the COVID-19 pandemic, the procedure of endodontics undergraduate lesson in Jinan university has a certain degree of interference. Although offline teaching has been basically resumed, the closure of the school means that students must seek permission from their counsellor to leave campus when necessary.

To the best of our knowledge, limited research has been conducted to compare the differences in learning attitudes and performance between mainland Chinese and non-mainland Chinese students towards the changes in dental education during the COVID-19 pandemic. This study aimed to assess the learning attitudes and outcomes of endodontics among mainland Chinese and non-mainland Chinese students during the pandemic. Additionally, the study seeks to investigate how economic, social, cultural, and educational backgrounds may influence these outcomes. The findings of this study may provide valuable insights for dental educators in planning and managing undergraduate dental education in the post-COVID-19 era.

## Methods

**Fig. 1:**
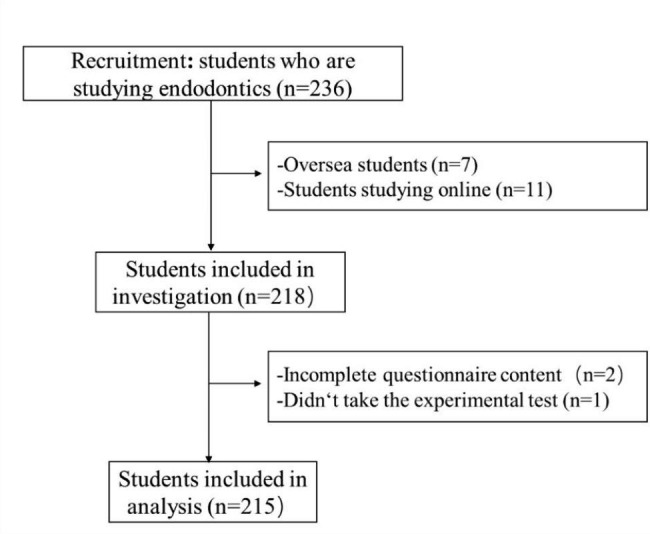
Flowchart of study inclusion

A cross-sectional study was conducted in November 2022 at the School of Stomatology, Jinan University in Guangzhou, Guangdong, China. Before this period, most areas in mainland China were still grappling with the COVID-19 pandemic. To prevent its spread, almost every university in China implemented measures to restrict students’ movement. In mainland China, the field of dentistry is commonly referred to as stomatology, and dental students are required to complete a five-year undergraduate program. The study targeted senior stomatology students eligible to take the endodontic course, including both mainland Chinese and non-mainland Chinese students. G-power software (version 3.1.9.7) was employed to perform the calculations to compare the means of two independent samples. The ρ and α values were determined based on the students’ scores from the previous year. It was estimated that a sample size of at least 67 participants in each group would be necessary to obtain meaningful results. A total of 215 participants completed the survey (Fig. 1). The study was approved by the ethics committee of Jinan University (JNUKY-2022-002), and participants provided e-written consents before responding to the questions.

### Changes in endodontics education during the COVID-19 pandemic

In most mainland Chinese schools of stomatology, the cariology, operative dentistry and endodontics are incorporated to a comprehensive course for senior students. Particularly, Jinan University’s stomatology program follows the western education system, where endodontics is an independent course in the fourth year, comprising both theoretical and skill sessions. With the outbreak of the COVID-19 pandemic, various digital tools such as PowerPoint lectures, video meetings, and massive online open courses were utilized for online teaching. In recent two years, offline teaching of endodontics has been basically resumed in Jinan University. Senior students, including mainland Chinese and non-mainland Chinese students, have been able to attend theoretical lessons in classrooms and skill sessions in head-simulator laboratories while wearing masks. Nevertheless, the campus has been closed and students were not allowed out unless getting permission from their counselor due to strong reasons. Moreover, a planned medical intern in department of endodontics at the end of the course was cancelled, depriving students of the chance to observe the actual endodontic treatment procedure.

### Questionnaire

During the final class of the endodontic course, students were requested to participate in an anonymous online questionnaire utilizing WeChat’s QuickMark code, a prevalent online survey platform in China. The questionnaire comprised of 22 questions, divided into three main categories. The first category focused on demographic characteristics, including two questions. The second category assessed perceptions and attitudes towards the endodontic course, encompassing seven questions including interest in endodontics, difficulty evaluation, teaching satisfaction, self-evaluation, among others. The third category evaluated perceptions and attitudes towards the impact of COVID-19 on the course, with 13 questions that addressed the influence of COVID-19 on the study of endodontics, mental health and social interaction, and attitudes towards COVID-19. The questionnaire drew on prior studies by Cheng et al. in Taiwan [17] and Bak et al. in the USA [18]. Respondents were instructed to choose only one level of agreement. All outcomes were self-reported through the questionnaire.

### Scores evaluation of the endodontic course

At the conclusion of the course, students were asked to participate in the assessment of both skill sessions and theoretical lessons and received their course marks. The content of the skill exam was the pulp opening of maxillary first molars. Students were graded based on how well they have done on the head-simulator according to a uniform standard by the same teacher. The score of pulp opening was divided into three parts, which were 30 points for specific operation (5 points for correct grip method of dental handpiece, 5 points for the stability of the fulcrum, 5 points for the selection of the drill needle, 5 points for the direction of the drill needle, 5 points for the intermittent grinding, and 5 points for the correct opening position), 20 points for morphology of pulp opening cavity (15 points for the correct hole type and 5 points for the smooth hole edge line), and 50 points for pulp opening effect (10 points for exposing all root canal orifices, 10 points for forming straight path, 10 points for uncovering the top of pulp chamber, 10 points for complete pulp chamber bottom, and 10 points for not forming steps). The theoretical exam evaluated students’ memory, comprehension, and application of related endodontic knowledge, comprising single choice (20 points), multiple choice (10 points), gap fill (10 points), noun interpretation (15 points), and essay questions (45 points). This study adopted a double-blind method. The two groups of students (mainland Chinese and non-mainland Chinese students) were mixed in class and assessment, and neither the recruited students nor teachers awarding the scores were aware of the groups’ identities.

### Statistical analysis

SPSS 22.0 (IBM Corp. New York, NY, USA) was used to analyze the Cronbach’s alpha coefficient. The content validity, clarity, and conciseness of the instrument were assessed by four experts, including one professor, one associate professor, and two lecturers [19]. Descriptive statistics were analyzed for the demographic variables, questionnaire, and skill and theoretical lessons scores. Continuous variables were presented as mean and standard deviation, while categorical variables were presented as cases (n) and percentage (%). Independent two-sample t-tests were used to compare continuous variables, while chi-square tests used for categorical variables between mainland Chinese and non-mainland Chinese students. Pearson correlation was conducted to describe the linear correlation between skill and theoretical scores by calculating Pearson correlation coefficient (r). All the statistical analysis was performed with SPSS 22.0 software, and a *P*-value of < 0.05 was considered statistically significant.

## Results

### Academic performance of students in the endodontic course

**Fig. 2:**
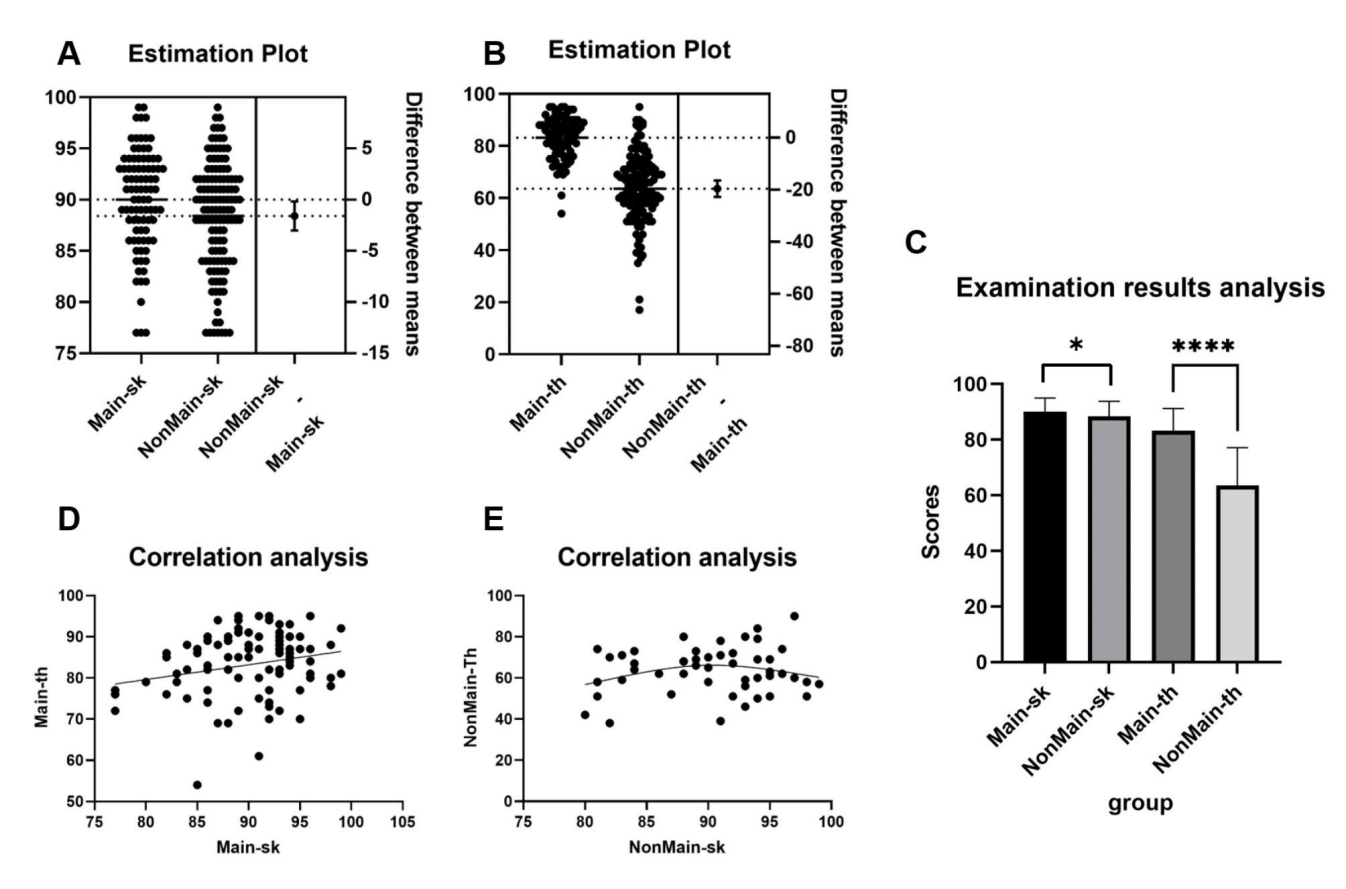
Test scores analysis for the endodontic course. Grade-point distribution of skill sessions (A). Grade-point distribution of theoretical lessons (B). Average score analysis of skill sessions and theoretical lessons (C). Correlation analysis of skill sessions and theoretical lessons (D, E). Main-sk and Main-th means the skill and theoretical scores of mainland Chinese students. NonMain-sk and NonMain-th means the skill and theoretical scores of students from Hong Kong, Macao, and Taiwan. *P < 0.05, ****P < 0.0001

A total of 215 students were recruited, including 89 mainland Chinese students and 126 non-mainland Chinese students. The scores of all students’ skill sessions were relatively scattered, while those of theoretical lessons were more tightly concentrated (Figs. 2A&B). Notably, both the skill and theoretical scores of mainland Chinese students were significantly higher than those of non-mainland Chinese students (skill: 90.0 ± 4.91 vs. 88.4 ± 5.38, *P* = 0.0265; theoretical: 83.2 ± 8.00 vs. 63.6 ± 13.5, *P* < 0.0001) (Fig. 2C). Of particular concern was the finding that 44 (34.9%) of non-mainland Chinese students failed to achieve the minimum passing grade of 60 points (out of 100) in the theoretical lessons, compared to only 2 (2.2%) of mainland Chinese students who received scores below 60 points.

Our study revealed that a linear relationship (Pearson correlation coefficients, r = 0.22) existed between the theoretical and experimental scores of mainland Chinese students (Y = 0.2314*X + 42.68) (Fig. 2D). Higher experimental scores are associated with better theoretical scores. Conversely, no such linear relationship (r = 0.19) between the skill and theoretical scores of non-mainland Chinese students was observed (Fig. 2E). It was found an inverted-U shape correlation, whereby the theoretical class score initially increased with the skill score, but subsequently decreased. That was, stronger experimental operation ability among mainland Chinese students is linked to better theoretical knowledge mastery, while non-mainland Chinese students’ operating ability does not appear to be significantly related to their theoretical knowledge level.

### Students’ view and evaluation of the endodontic course

Reliability analysis of the post-course survey demonstrated strong internal consistency, with Cronbach’s alpha coefficients of 0.799. Additionally, a content validity index (CVI) was calculated for the questionnaire items, revealing high validity with both item-level CVI (I-CVI) and scale-level CVI (S-CVI) values of 1.

As shown in Table 1, in terms of gender composition, the number of female students recruited was significantly higher than male students (141, 65.6% and 74, 34.4%, respectively). The gender ratio was also significantly different between mainland Chinese and non-mainland Chinese students (*P* = 0.026). Overall, 86% of all dental students were interested or very interested in endodontics. More non-mainland Chinese students (84.1%) found the endodontic course difficult or very difficult compared to mainland students (73%) (*P* = 0.047). Nevertheless, most students from both groups (95%) agreed that skill sessions were very helpful for learning endodontics. Regarding students’ self-evaluation on their experimental operation and theoretical knowledge ability, 18 (20.2%) and 27 (30.3%) of mainland Chinese students, 43 (34.1%) and 43 (34.1%) of non-mainland Chinese students gave themselves excellent score (4 or 5 points). Non-mainland Chinese students seemed to be more confident and had a high self-evaluation in operation ability (*P* = 0.026).

**Table 1:** Perception and attitudes toward the endodontic course

	All n (%)	Mainland Chinese students n (%)	Non-Mainland Chinese students n (%)	*P*-value
**Gender**				
Male	74 (34.4)	23 (25.8)	51 (40.5)	0.026*
Female	141 (65.6)	66 (74.2)	75 (59.5)	
**1-1. Are you interested in endodontic?**				
very interested/ Interested	185 (86.0)	74 (83.1)	111 (88.1)	0.302
Uninterested/ No interest at all	30 (14)	15 (16.9)	15 (11.9)	
**1-2. How do you think the difficulty of the endodontic course?**				
Very difficult/ Difficult	171 (79.5)	65 (73.0)	106 (84.1)	0.047*
Not very difficult/ Simple	44 (20.5)	24 (27.0)	20 (15.9)	
**1-3. Do you agree that the experimental course is helpful for learning endodontics?**				
Strongly Agree/ Agree	208 (96.7)	85 (95.5)	123 (97.6)	0.638
Disagree/ Strongly Disagree	7 (3.3)	4 (4.5)	3 (2.4)	
**1-4. How do you think the score of your experimental operation ability is (5 points in full score)?**				
4 and 5 points	61 (28.4)	18 (20.2)	43 (34.1)	0.026*
≤ 3 points	154 (71.6)	71 (79.8)	83 (65.9)	
**1-5. How do you think the score of your theoretical knowledge ability is (5 points in full score)?**				
4 and 5 points	70 (32.6)	27 (30.3)	43 (34.1)	0.559
≤ 3 points	145 (67.4)	62 (69.7)	83 (65.9)	
**1-6. Are you satisfied with the current teaching method?**				
Very satisfied/ Satisfied	183 (85.1)	80 (89.9)	103 (81.7)	0.099
Dissatisfied/ Very Dissatisfied	31 (14.9)	9 (10.1)	23 (18.3)	
**1-7. Will you choose endodontics as the future development direction?**				
Definitely/ Very likely	59 (27.4)	24 (27.0)	35 (27.8)	0.179
Maybe	113 (52.6)	42 (47.2)	71 (56.3)	
Most likely not/ Definitely not	43 (20.0)	23 (25.8)	20 (15.9)	

Chi-square test; *P* < 0.05 values were significant (*P < 0.05)

Overall, more than 85% of all students were satisfied with the current teaching methods, with mainland Chinese students being slightly more satisfied (89.9%) than non-mainland Chinese students (81.7%). As for career choice, only 24 (27%) mainland Chinese students and 35 (27.8%) non-mainland Chinese education students expressed interest in choosing the endodontics as the future development direction, while 52.6% of students were neutral and had not determined their future employment direction.

### Students’ perception and attitudes on dental education under COVID-19 pandemic

Table 2 presents a comparison of the responses regarding dental education under the COVID-19 pandemic between mainland Chinese and non-mainland Chinese students. The questionnaire referred to a Cheng et al.’s study in Taiwan [17]. Our findings indicated significant differences in the proportion of “agree” responses for seven items between the two groups. Non-mainland Chinese students exhibited a higher degree of agreement with items such as “I think that the COVID-19 impact my choice of selecting endodontics as a future employment direction”, “I think that the COVID-19 affect me to learn endodontics”, “I think that the COVID-19 has an impact on the development of endodontics”, “The COVID-19 affected my original plans for choosing a career in the future”, “The COVID-19 changed my personal hygiene”, “I’m worried about being infected with COVID-19”, and “The COVID-19 has a greater impact on my learning emotions”. These results showed that the COVID-19 pandemic has had a more pronounced effect on non-mainland Chinese students than that on mainland Chinese students.

**Table 2:** Perception and attitudes toward the impact of COVID-19

Items	All n (%)	Mainland Chinese students n (%)	Non-Mainland Chinese students n (%)	*P*-value
**Endodontic study**				
**2-1. I think that the COVID-19 impact my choice of selecting endodontics as a future employment direction**				
Strongly Agree/ Agree	119 (55.3)	37 (41.7)	82 (65.1)	0.001**
Disagree/Strongly Disagree	96 (44.7)	52 (58.3)	44 (34.9)	
**2-2. I think that the COVID-19 affect me to learn endodontics**				
Strongly Agree/ Agree	81 (37.7)	25 (28.1)	56 (44.4)	0.015*
Disagree/Strongly Disagree	134 (62.3)	64 (71.9)	70 (55.6)	
**2-3. I think that the COVID-19 has an impact on the development of endodontic**				
Strongly Agree/ Agree	143 (66.5)	47 (52.8)	96 (76.2)	< 0.001***
Disagree/Strongly Disagree	72 (33.5)	42 (47.2)	30 (23.8)	
**Mental Health and knowledge**				
**2-4. I’m pessimistic about the development of COVID-19**				
Strongly Agree/ Agree	109 (50.7)	39 (43.8)	70 (55.6)	0.09
Disagree/Strongly Disagree	106 (49.3)	50 (56.2)	56 (44.4)	
**2-5. I’m actively gather the latest medical information about COVID-19 and in-depth knowledge**				
Strongly Agree/ Agree	127 (59.1)	53 (59.6)	74 (58.7)	0.904
Disagree/Strongly Disagree	88 (40.9)	36 (40.4)	52 (41.3)	
**Social Network and Behavior**				
**2-6. The COVID-19 affected my original plans for choosing a career in the future**				
Strongly Agree/ Agree	100 (46.5)	34 (38.2)	66 (52.4)	0.04*
Disagree/ Strongly Disagree	115 (53.5)	55 (61.8)	60 (47.6)	
**2-7. The COVID-19 changed my social interaction**				
Strongly Agree/ Agree	187 (87.0)	74 (83.1)	113 (89.7)	0.161
Disagree/Strongly Disagree	28 (13.0)	15 (16.9)	13 (10.3)	
**2-8. The COVID-19 changed my way of relaxing**				
Strongly Agree/ Agree	173 (80.5)	67 (75.3)	106 (84.1)	0.107
Disagree/Strongly Disagree	42 (19.5)	22 (24.7)	20 (15.9)	
**2-9. The COVID-19 changed my personal hygiene**				
Strongly Agree/ Agree	168 (78.1)	62 (69.7)	106 (84.1)	0.011*
Disagree/Strongly Disagree	47 (21.9)	27 (31.3)	20 (15.9)	
**Attitudes**				
**2-10. I’m worried about being infected with COVID-19**				
Strongly Agree/Agree	122 (56.7)	43 (48.3)	79 (62.7)	0.036*
Disagree/Strongly Disagree	93 (43.3)	46 (51.7)	47 (37.3)	
**2-11. I’m worried that COVID-19 will continue**				
Strongly Agree/Agree	170 (79.1)	73 (82.0)	97 (77.0)	0.371
Disagree / Strongly Disagree	45 (20.9)	16 (18.0)	29 (23.0)	
**2-12. The COVID-19 has a greater impact on my learning emotions**				
Strongly Agree/ Agree	137 (63.7)	47 (52.8)	90 (71.4)	0.005**
Disagree/ Strongly Disagree	78 (36.3)	42 (47.2)	36 (28.6)	
**2-13. I’m worried that COVID-19 create financial pressure for my school studies**				
Strongly Agree/ Agree	140 (65.1)	53 (59.6)	87 (69.0)	0.15
Disagree/Strongly Disagree	75 (34.9)	36 (40.4)	39 (31.0)	

Chi-square test; *P* < 0.05 values were significant (*P < 0.05, **P < 0.01, ***P < 0.001)

Furthermore, both groups of students strongly agreed that the COVID-19 pandemic has altered their social interactions and leisure activities and expressed concerns about the ongoing impact of the pandemic on their financial situation and campus studies.

## Discussion

With the acceleration of the Greater Bay Area (Guangdong-Hong Kong-Macao) integration process and the continuous improvement of the education level in mainland Chinese, an increasing number of students from Hong Kong, Macao, Taiwan, and even overseas have opted to pursue higher education in mainland China [20]. In this study, we conducted a cross-sectional survey in Jinan University to evaluate the learning attitudes and outcomes of dental students in endodontics during the COVID-19 pandemic. Our findings indicated that non-mainland Chinese students received lower grades and were more adversely affected by COVID-19 than mainland Chinese students, in terms of their mental well-being.

Jinan University has the largest number of non-mainland Chinese students. To cater to the different capabilities and needs of two types to students, group teaching has been implemented in some courses. However, the endodontic course is not included. In this study, both mainland Chinese and non-mainland Chinese students received the same theoretical and skill lessons of endodontics. The results showed that mainland Chinese students performed significantly better than non-mainland Chinese students in the course. This difference could be attributed to the varying educational systems (primary and secondary education) between mainland China and Hong Kong, Macao along with Taiwan, including differences in curricula, teaching methods, education programs, facilities and more [20]. The education systems in Hong Kong, Macao, and Taiwan reflect a combination of Chinese and Western influences and share more similarities with those in western developed countries [14, 21]. Our study also found that non-mainland Chinese students had lower satisfaction levels with the current teaching methods compared to mainland Chinese students, although the difference was not statistically significant (81.7% vs. 89.9%). Therefore, the poorer academic performance of non-mainland Chinese students in endodontics may be attributed to their difficulties in adapting to the education system in mainland China.

Undergraduate courses of dentistry have been acknowledged as unique due to the substantial amount of hands-on training included in skill sessions [22]. As reported, there exists a considerable divergence among dental schools regarding the time dedicated to theoretical and skills instructions in endodontics [16]. In this study, the theoretical and skill scores of mainland Chinese students were directly proportional, suggesting that engaging experimental operation was conducive to improving theoretical knowledge level in endodontics. Nevertheless, for non-mainland Chinese students, an increase in skill sessions scores corresponded initially with an increase in theoretical lessons scores, which later decreased. Moreover, 34.9% of non-mainland Chinese students failed to reach the pass mark in theoretical lessons, and a higher proportion of these students found the course challenging compared to mainland Chinese students. These findings demonstrated that while non-mainland Chinese students have strong operation abilities, their operational and theoretical learning did not align effectively. As such, it is crucial to improve their efficiency of theoretical study urgently.

In this study, over 85% dental students were interested in endodontics and satisfied with the current teaching method. This finding is consistent with two recent studies conducted in UK dental schools, which also showed that many students were satisfied with the amount of time spent on endodontic teaching and the quality of teaching [23, 24]. Despite this, more than 80% of students found the endodontic course difficult. For endodontic educators, it is imperative to improve teaching methods to help students elevate the efficiency and quality of learning. The usage of mind maps in dental education is in favor of students with different learning styles and help the instructor to identify the level of conceptualization [25]. Improvements suggested for future endodontic education also include student-oriented inducing mode, problem-based learning, and strengthened student supervision.

The COVID-19 pandemic has led to remarkable changes in campus life and study. While staying at home and avoiding gatherings and travel can help reduce the risk of infection and transmission, it has also limited communication and social activities, leading to economic downturns in many countries [2]. In this study, 65.1% of dental students expressed concern about the financial pressure caused by the pandemic on their school life. This percentage is higher than that reported in similar studies conducted in Poland and the USA, which reported rates of 48.7% and 59.2%, respectively [26, 27]. Additionally, 46.5% of the students in our study indicated that their original plans for their future careers were affected by the pandemic. This finding is consistent with a previous study conducted at the University of Vermont (in USA), whereas 74.5% of students expressed concern about their career development [26]. Hence, it is essential to pay more attention to the impact of the COVID-19 pandemic on students’ career choices and confidence.

Aside from financial pressure and career planning, the most serious impact is the mental health of students during the COVID-19 pandemic. Numerous studies have shown that the pandemic has increased symptoms of depression and mood disorders, especially among those with pre-existing mental or physical health problems, those living in deprived areas, and ethnic minority communities [28–30]. A study conducted at the University of Zagreb (in Croatia) found that 66.2% of college students were psych-emotionally affected by the lockdown [31]. The imposition of public health measures, such as social distancing and lockdown, social fear related to COVID-19, fear of being infected, and anxiety for their removal from clinical practice, all had negative effects on the psychological well-being of medical students [9]. Consequently, a high prevalence of moderate depression, anxiety, and stress had been reported among medical students during the COVID-19 pandemic [32]. A previous study in the Western University of Health Sciences (in USA) demonstrated that 55.6% of dental students felt depressed during the pandemic and most dental students (79.2%) had not received counseling or seen a therapist even once [27]. Moreover, in this study, 63.7% of dental students agreed that the pandemic had impacted their learning emotional state due to changes in social contact, entertainment, and concerns about infection, financial condition, and career prospect, etc. There findings highlight the urgent need to pay attention to students’ psychological health and provide them positive support and encouragement. Particularly, more and more women have chosen dentistry as their career path in recent years [33, 34], and female students accounted for 65.6% of dental seniors in our study. Females and those infected with COVID-19 infection had higher rates of almost all outcomes related to mental health [35]. Thus, it is imperative to prioritize the mental health of female dental students.

Our study revealed that the COVID-19 pandemic had a great impact on non-mainland Chinese students’ emotions compared to the mainland Chinese students. The outbreak of COVID-19 significantly reduced international student mobility [36], leading to over 300 universities in mainland China enrolling new students from Hong Kong, Macao, and Taiwan in 2020 [37]. These enrollments may have included students who had originally intended to study abroad. Our findings showed that non-mainland Chinese students experienced more emotional distress related to their study, campus life, and future career choices. Previous study has also demonstrated that international students studying outside their hometowns were at a higher risk for developing anxiety disorders during collective trauma such as the pandemic [38]. During the pandemic, non-mainland Chinese students faced virous obstacles when returning home, which likely contributed to higher levels of anxiety and maladjustment compared to mainland Chinese students. Therefore, it is crucial to provide additional support services for non-mainland Chinese students and other international students, such as online support groups and narrative therapy, to alleviate their fears and anxieties.

However, limitations still existed in our study. Firstly, we conducted our study at a single university, and our results may not be completely generalizable to other campuses. Secondly, our study relied on self- reported online questionnaires, which are vulnerable to self- reported biases. Finally, we did not collect identifying information about individual students, which limits our ability to correlate responses with their academic performance.

## Conclusions

In the endodontic course, non-mainland Chinese students demonstrated poorer performance compared to mainland Chinese students. The COVID-19 pandemic had a greater impact on the metal well-being of non-mainland Chinese students in terms of learning emotions, personal hygiene, and future career choices, potentially affecting their academic grades. Dental educators should consider the diversity of their students. To achieve a sense of “normality” once again, dental school may need to implement significant changes to enhance the mental health and academic achievements of their students, particularly non-mainland Chinese and international students.

## Data Availability

We did not share our data for the disclosure of students’ materials. The datasets used and/or analyzed during the current study are available from the corresponding author (imshihs@jnu.edu.cn) on reasonable request.

## Abbreviations

COVID-19: Coronavirus disease-19
CVI: Content validity index

## References

[1] Weekly epidemiological update on COVID-19–11 January 2023: World Health Organization (WHO). ; 2023 [Available from: https://www.who.int/publications/m/item/weekly-epidemiological-update-on-covid-19---11-january-2023.

[2] Chang T-Y, Hong G, Paganelli C, Phantumvanit P, Chang W-J, Shieh Y-S (2021). Innovation of dental education during COVID-19 pandemic. J Dent Sci.

[3] Li Y, Chai Y, Chen Z, Li C (2023). From lockdown to precise prevention: adjusting epidemic-related spatial regulations from the perspectives of the 15-minute city and spatiotemporal planning. Sustain Cities Soc.

[4] Wang W, Wang J, Zhang X, Pei Y, Tang J, Zhu Y (2023). Network connectivity between anxiety, depressive symptoms and psychological capital in chinese university students during the COVID-19 campus closure. J Affect Disord.

[5] Bai Y, Ma S (2023). From depression to wellbeing: how to protect the mental health of isolated people under the “dynamic clearance” of patients with COVID-19. Front Psychol.

[6] Reimers FM (2022). Learning from a pandemic. The impact of COVID-19 on education around the world. Primary and secondary education during COVID-19.

[7] Li L, Wu B, Patwary AK (2022). The psychosocial factors affecting chinese Outbound Exchange and mobility students’ academic performance during COVID-19. Front Psychol.

[8] Papapanou M, Routsi E, Tsamakis K, Fotis L, Marinos G, Lidoriki I (2022). Medical education challenges and innovations during COVID-19 pandemic. Postgrad Med J.

[9] Vala NH, Vachhani MV, Sorani AMJNJoP (2020). Pharmacy, Pharmacology. Study of anxiety, stress, and depression level among medical students during COVID-19 pandemic phase in Jamnagar city. Natl J Physiol Pharm Pharmacol.

[10] Goriuc A, Sandu D, Tatarciuc M, Luchian IJIJoER, Health P (2022). The impact of the COVID-19 pandemic on dentistry and dental education: a narrative review. Int J Environ Res Public Health.

[11] Wang T. How to Build World-Class Universities in Guangdong-Hong Kong-Macao Greater Bay Area? Higher Education, Innovation and Entrepreneurship from Comparative Perspectives: Springer Nature Singapore. 2022. p. 165 – 84.

[12] Seah WT, Zhang Q, Barkatsas T, Law HY, Leu Y-CJNACotIGftPoME. Mathematics Learning in Mainland China, Hong Kong and Taiwan: the values perspective. North American Chapter of the International Group for the Psychology of Mathematics Education; 2014.

[13] Yu B, Wright EJTE (2017). Management. Academic adaptation amid internationalisation: the challenges for local, mainland chinese, and international students at Hong Kong’s universities. Tert Educ Manag.

[14] Yu B, Mak AS, Bodycott PJSiHE (2021). Psychological and academic adaptation of mainland chinese students in Hong Kong universities. Stud High Educ.

[15] De Moor R, Hülsmann M, Kirkevang LL, Tanalp J, Whitworth J (2013). Undergraduate curriculum guidelines for endodontology. Int Endod J.

[16] Sacha SR, Sonntag D, Burmeister U, Rüttermann S, Gerhardt-Szép S (2021). A multicentric survey to evaluate preclinical education in Endodontology in German-speaking countries. Int Endod J.

[17] Cheng H-C, Lu S-L, Yen Y-C, Siewchaisakul P, Yen AM-F, Chen SL-S (2021). Dental education changed by COVID-19: Student’s perceptions and attitudes. BMC Med Educ.

[18] Bak S-Y, Saglik B, Inglehart MR. Introducing dental students to complete denture treatment in times of COVID-19: Students’ responses. J Dent Educ. 2022. 10.1002/jdd.13117 36251354

[19] Polit DF, Beck CT, Owen SV (2007). Is the CVI an acceptable indicator of content validity? Appraisal and recommendations. Res Nurs Health.

[20] Hu Y, Liu CJOJoSS (2022). Analysis of the demands, Dilemmas, and Paths of Basic Education Development in the Guangdong-Hong Kong-Macao Greater Bay Area. Open J Social Sci.

[21] Liang Q, Trends, Features and Logics of Policy Changes on Higher Education Cooperation in Guangdong-Hong Kong-Macau. Analysis based on 65 related policy texts. Higher Education, Innovation and Entrepreneurship from comparative perspectives. Springer; 2022. pp. 109–38.

[22] Lin GSS, Tan WW, Chan DZK, Chua KH, Yee TC, Lazaldin MAM (2022). Quality of endodontic record-keeping and root canal obturation performed by final year undergraduate dental students: an audit during the COVID-19 pandemic. PLoS ONE.

[23] Al Raisi H, Dummer P, Vianna MJIej (2019). How is Endodontics taught? A survey to evaluate undergraduate endodontic teaching in dental schools within the United Kingdom. Int Endod J.

[24] Puryer J, Amin S, Turner M. Undergraduate Confidence When Undertaking Root Canal Treatment and Their Perception of the Quality of Their Endodontic Education. Dent J (Basel). 2016;5(1). 10.3390/dj5010001 PMC5806992 29563408

[25] Grazziotin-Soares R, Curtis DA, Ardenghi DM (2021). Use of mind maps in dental education: an activity performed in a preclinical endodontic course. J Dent Educ.

[26] Sauer N, Sałek A, Szlasa W, Ciecieląg T, Obara J, Gaweł S et al. The Impact of COVID-19 on the Mental Well-Being of College Students. Int J Environ Res Public Health. 2022;19(9). 10.3390/ijerph19095089 PMC9100955 35564484

[27] Hayes C, Mears M, Rowan S, Dong F, Andrews E (2022). Academic performance and attitudes of dental students impacted by COVID-19. J Dent Educ.

[28] Robinson E, Sutin AR, Daly M, Jones A (2022). A systematic review and meta-analysis of longitudinal cohort studies comparing mental health before versus during the COVID-19 pandemic in 2020. J Affect Disord.

[29] Weich S (2022). Mental health after covid-19. BMJ.

[30] The Lancet P (2021). COVID-19 and mental health. Lancet Psychiatry.

[31] Badovinac A, Par M, Plančak L, Balić MD, Vražić D, Božić D et al. The Impact of the COVID-19 Pandemic on Dental Education: An Online Survey of Students’ Perceptions and Attitudes. Dent J (Basel). 2021;9(10). 10.3390/dj9100116 PMC8535140 34677178

[32] Paz DC, Bains MS, Zueger ML, Bandi VR, Kuo VY, Cook K (2022). COVID-19 and mental health: a systematic review of international medical student surveys. Front Psychol.

[33] Markvart M, Fransson H, Bjørndal L (2018). Ten-year follow-up on adoption of endodontic technology and clinical guidelines amongst danish general dental practitioners. Acta Odontol Scand.

[34] Haug SR, Linde BR, Christensen HQ, Vilhjalmsson VH, Bårdsen A (2021). An investigation into security, self-confidence and gender differences related to undergraduate education in endodontics. Int Endod J.

[35] Dragioti E, Li H, Tsitsas G, Lee KH, Choi J, Kim J (2022). A large-scale meta-analytic atlas of mental health problems prevalence during the COVID-19 early pandemic. J Med Virol.

[36] Mok KH, Xiong W, Ke G, Cheung JOWJIJoER (2021). Impact of COVID-19 pandemic on international higher education and student mobility: student perspectives from mainland China and Hong Kong. Int J Educational Res.

[37] Wen L. Innovative Mode of Education for College Students Majoring in Chinese from Hong Kong, Macao, Taiwan and Overseas Through Practical Education. International Conference on Education Studies: Experience and Innovation (ICESEI 2020). Atlantis Press, 2020: 452–461.

[38] Feng S, Zhang Q, Ho SMY (2021). Fear and anxiety about COVID-19 among local and overseas chinese university students. Health Soc Care Community.

